# Immunotoxicity Assessment of Rice-Derived Recombinant Human Serum Albumin Using Human Peripheral Blood Mononuclear Cells

**DOI:** 10.1371/journal.pone.0104426

**Published:** 2014-08-06

**Authors:** Kai Fu, Qin Cheng, Zhenwei Liu, Zhen Chen, Yan Wang, Honggang Ruan, Lu Zhou, Jie Xiong, Ruijing Xiao, Shengwu Liu, Qiuping Zhang, Daichang Yang

**Affiliations:** 1 State Key Laboratory of Hybrid Rice and College of Life Sciences, Wuhan University, Wuhan, China; 2 Department of Immunology, College of Basic Medical Science, Wuhan University, Wuhan, China; 3 Institute of Hydrobiology, Chinese Academy of Sciences, Analysis and Testing center, Wuhan, China; University of California, San Francisco, United States of America

## Abstract

Human serum albumin (HSA) is extensively used in clinics to treat a variety of diseases, such as hypoproteinemia, hemorrhagic shock, serious burn injuries, cirrhotic ascites and fetal erythroblastosis. To address supply shortages and high safety risks from limited human donors, we recently developed recombinant technology to produce HSA from rice endosperm. To assess the risk potential of HSA derived from *Oryza sativa* (OsrHSA) before a First-in-human (FIH) trial, we compared OsrHSA and plasma-derived HSA (pHSA), evaluating the potential for an immune reaction and toxicity using human peripheral blood mononuclear cells (PBMCs). The results indicated that neither OsrHSA nor pHSA stimulated T cell proliferation at 1x and 5x dosages. We also found no significant differences in the profiles of the CD4^+^ and CD8^+^ T cell subsets between OsrHSA- and pHSA-treated cells. Furthermore, the results showed that there were no significant differences between OsrHSA and pHSA in the production of cytokines such as interferon-gamma (IFN-γ), tumor necrosis factor-alpha (TNF-α), interleukin (IL)-10 and IL-4. Our results demonstrated that OsrHSA has equivalent immunotoxicity to pHSA when using the PBMC model. Moreover, this *ex vivo* system could provide an alternative approach to predict potential risks in novel biopharmaceutical development.

## Introduction

Plant-made pharmaceuticals (PMPs) are a category of therapeutic products based on a plant platform that has developed rapidly over the past two decades [1]. Up until now, plant cells have successfully been used to produce at least 108 pharmaceuticals; more than 30 PMP products have been evaluated in clinical trials, and nine have been approved for market [2]. Various plant species, including rice, tobacco, maize, soybean, potato, barley, carrot and safflower, have been tested to produce various antibodies, cytokines, vaccines and enzymes [3]. Currently, rice seeds produce many recombinant pharmaceutical proteins, including cholera toxin B subunit [4], human transferrin [5], human serum albumin [6], human α-antitrypsin [7] and human basic fibroblast growth factor [8]. Rice endosperm is thought to be an excellent bioreactor for PMP production [2].

Over the past decades, attempts to produce rHSA in various expression systems have been made, including *Escherichia coli* [9], *Saccharomyces cerevisiae* [10], *Kluyveromyces lactis* [11], *Pichia pastoris* [12], transgenic animals [13], and transgenic plants [6], [14], [15], [16], [17]. Although rHSA has been successfully expressed in these systems, only a few rHSA have proceeded to clinical trials. The first rHSA (Medway produced by Mitsubishi Pharma Corporation, Japan) has been entered clinical trial. The results have showed that no serious allergy or difference in the incidence of adverse drug reaction (ADRs) was observed. However, allergic ADRs were observed, while the specific IgE antibodies were not detected [18]. Recently, the clinical trial of rHSA (Recombumin produced by Biopharma-Novozymes, UK) has been conducted. The results have indicated that intramuscular and intravenous administration of Recombumin to be well tolerated with no treatment-related serious adverse events, and no evidence of an immunologic response [19].

Because the plant system is a novel method of biopharmaceutical production, several methodologies could be proposed for evaluating the preclinical safety of PMPs. However, biopharmaceuticals derived from different biological sources could have diverse components in their manufacturing processes compared with conventional small molecule chemical drugs [20]. Therefore, a safety assessment using appropriate animal systems is an essential step before a novel biopharmaceutical is approved for clinical trial. In spite of stringent safety assessments for the use of novel biopharmaceuticals in animal systems, a potential risk still exists. For example, in 2006, six healthy volunteers all suffered a series of serious adverse reactions and life-threatening inflammatory responses from a “cytokine storm” after injection of a 1/500 dose of the anti-CD28 monoclonal antibody TGN1412, which was found to be safe in animal tests [21], [22]. This incident justifies the need for predicting the potential immune effects of a biopharmaceutical before the investigational new drug (IND) moves forward to clinical trials.

Recently, an *ex vivo* assay based on human peripheral blood mononuclear cells (hPBMCs) or other cell lines has been used to predict the species-specific activity and immunotoxicity of biopharmaceuticals in preclinical safety testing [23]. These cell-based assays are more closely related to human immunological systems, which could be an appropriate *in vitro* assessment. Based on those rationales, regulatory agencies recommend and encourage manufacturers to use *in vitro* or *ex vivo* assessments to predict the immune effects during the preclinical safety evaluation of biopharmaceuticals [20]. PBMCs have been widely used in many fields, such as immunology, infectious diseases, hematological malignancies, vaccine development, transplant immunology and high-throughput screening for drug candidates [24]. Stebbings et al. have reported that PBMCs could efficiently predict the species-specific activity, immunogenicity or immunotoxicity of monoclonal antibodies (mAbs) and vaccines in preclinical safety testing [23], [25]. Furthermore, PBMCs have also been successfully used for monitoring the effects of mAb complexes on the innate immune response *in vitro* [26]. In addition, PBMCs have been efficiently used to evaluate the safety and immunogenicity of booster vaccinations [27], [28], [29]. Moreover, a cytokine release assay (CRA) with human PBMCs has also been used as an alternative tool to predict the immunotoxicities of monoclonal antibodies and vaccines [30]. Therefore, PBMCs could be used in alternative approaches to predict the potential for immunotoxicity of PMP products.

In this study, we used PBMCs as an *ex vivo* system for the assessment of the risk potential for immunoreactions with recombinant human serum albumin derived from rice endosperm (OsrHSA). We investigated T cell proliferation and the profiles of T cell subsets, especially CD8^+^ cytotoxic T cells, along with cytokine release from cells treated with OsrHSA [6]. Our results indicated that both OsrHSA and plasma-derived human serum albumin (pHSA) could not stimulate T cell proliferation at 1x and 5x dosages. No differences in T cell phenotypes were observed between OsrHSA- and pHSA-treated cells. The secretion levels of four cytokines related to inflammation and immune regulation, including interferon-gamma (IFN-γ), tumor necrosis factor-alpha (TNF-α), interleukin (IL)-10 and IL-4, were not significantly different between OsrHSA and pHSA treatments. Our results demonstrated that OsrHSA is equivalent to pHSA in terms of its effects on T cell proliferation, CD4^+^ and CD8^+^ T cells and cytokine levels, suggesting that there is no evident potential for immunotoxicity with OsrHSA treatment in PBMC.

## Materials and Methods

### Reagents and controls

OsrHSA (HSA purity >99.99%) was purchased from Healthgen Biotechnology Co., Ltd., Wuhan, China. pHSA (HSA purity >96%) for a parallel control was purchased from the Wuhan Institute of Biological Products, Wuhan, China. PBS (0.0067 M PO_4_, pH 7.2) purchased from HyClone, Utah, USA, and PHA from Sigma-Aldrich, St. Louis, MO, USA, were used as the negative and positive controls, respectively.

### Blood samples and ethics statement

All blood samples (n = 20) were acquired from Wuhan Blood Center and approved by Blood Administration Center of Hubei Province. The studies were performed at the Medical School of Wuhan University under the guidance of the physician.

### Experimental design

All experiments were designed as described in Table 1. The dosage of HSA was calculated based on the clinical dosage of HSA and the blood volume of normal human adults (approximately 5,000 ml). The concentrations of pHSA and OsrHSA designated as the 1x and 5x dosages were calculated from a 1x clinical dosage of 10 g/5000 ml, or 2 mg/ml, HSA, which is equivalent to the 1x clinical dosage of 10 g/60 kg body weight. Three replicates were performed for each dosage. To eliminate the individual differences between the 1x and 5x dosages, volunteers were divided into two groups for testing the different doses of pHSA and OsrHSA. All measurements were taken at 24, 48 and 72 h for PBMC cultures, based on the optimization of the conditions in the preliminary experiments (Table 1).

**Table 1:** The dosage and time course of the PHA, PBS, pHSA and OsrHSA treatments given to PBMCs.

	PHA	PBS	pHSA		OsrHSA	
**Dosage**	10 µg/ml	NC	1x	5x	1x	5x
**Volunteers**	20	20	10	10	10	10

NC denotes negative control.

### PBMC preparation and isolation

PBMCs were isolated from the plasma by density gradient centrifugation using Ficoll-Paque Plus (GE Healthcare, Uppsala, Sweden) according to the manufacturer’s instructions. The PBMCs were suspended in a culture medium consisting of RPMI 1640 medium with L-glutamine, 10% heat-inactivated fetal bovine serum (FBS, HyClone, Utah, USA) and 0.5% penicillin/streptomycin solution (1x, HyClone, Utah, USA). The viability of the cells was determined by Trypan blue staining. Approximately 3–4×10^7^ PBMCs with over 95% viability were obtained from 100 ml plasma from each donor. The PBMCs were immediately used for future study.

### T cell proliferation assay

The T cell proliferation assay was performed using CCK-8 (Dojindo, Kumamoto, Japan) and CFSE (BioLegend, San Diego, CA) kits following the manufacturer’s instructions. For CCK-8 determination, approximately 2×10^5^ PBMCs per well were seeded in 100 µl medium in a 96-well microtiter plate and cultured in a CO_2_ incubator at 37°C with 5% CO_2_. At the time course endpoints (24, 48 and 72 h), 10 µl of the CCK-8 solution was added to the wells containing the PBMCs, and the plate was incubated at 37°C for 4 h. T cell proliferation was measured with a microtiter plate reader (VICTOR X5, Perkin Elmer, Waltham, MA, USA). For the CFSE assay, approximately 4×10^5^ PBMCs per well were labeled with 3 µM CFSE at 37°C for 5 min and then seeded in 500 µl medium in a 24-well microtiter plate. The T lymphocyte proliferation at each time point was evaluated at 488 nm using a BD FACS Aria II flow cytometer (BD Bioscience, San Jose, CA, USA).

### Determination of T cell phenotypes

The T cell phenotypes were determined using a BD FACSAria II flow cytometer. Approximately 5×10^5^ PBMCs per well were seeded in 1 ml medium in a 12-well microtiter plate and then treated as shown in Table 1. Before measurement, the PBMCs were washed with PBS once and then stained with CD3-phycoerythrin (PE), CD4-fluorescein isothiocyanate (FITC) and CD8-allophycocyanin (APC) in the dark at room temperature for 30 min. The cells were measured by setting a primary gate for lymphocytes, followed by CD3^+^/CD4^+^ and CD3^+^/CD8^+^.

### Cytokine release assay

Approximately 2×10^5^ PBMCs per well were seeded in 100 µl medium in a 96-well microtiter plate and were then treated as shown in Table 1. The supernatants were collected by centrifugation at 300×*g* for 5 min. The expression levels of cytokines released in the supernatant were determined using a BD FACSAria II flow cytometer using a BD CBA (Cytometric Bead Array) kit as described in the manufacturer’s instructions (BD Bioscience, San Jose, CA, USA). Data were calculated using the FACP Array software (BD Bioscience, San Jose, CA, USA).

### Data analysis

All data were processed using GraphPad Prism 5 software (GraphPad Software, La Jolla, CA, USA). Statistical analysis was performed with Student’s *t*-test. For cytokine release data, a hierarchical cluster analysis (HCA) was performed to compare the global response as previously described [31]. Clusters were defined by Euclidian distance and average linkage using IBM SPSS (Statistical Package for the Social Sciences) 19.0 software (IBM Corporation, Armonk, New York, USA).

## Results

### OsrHSA does not stimulate T cell proliferation

**Figure 1:**
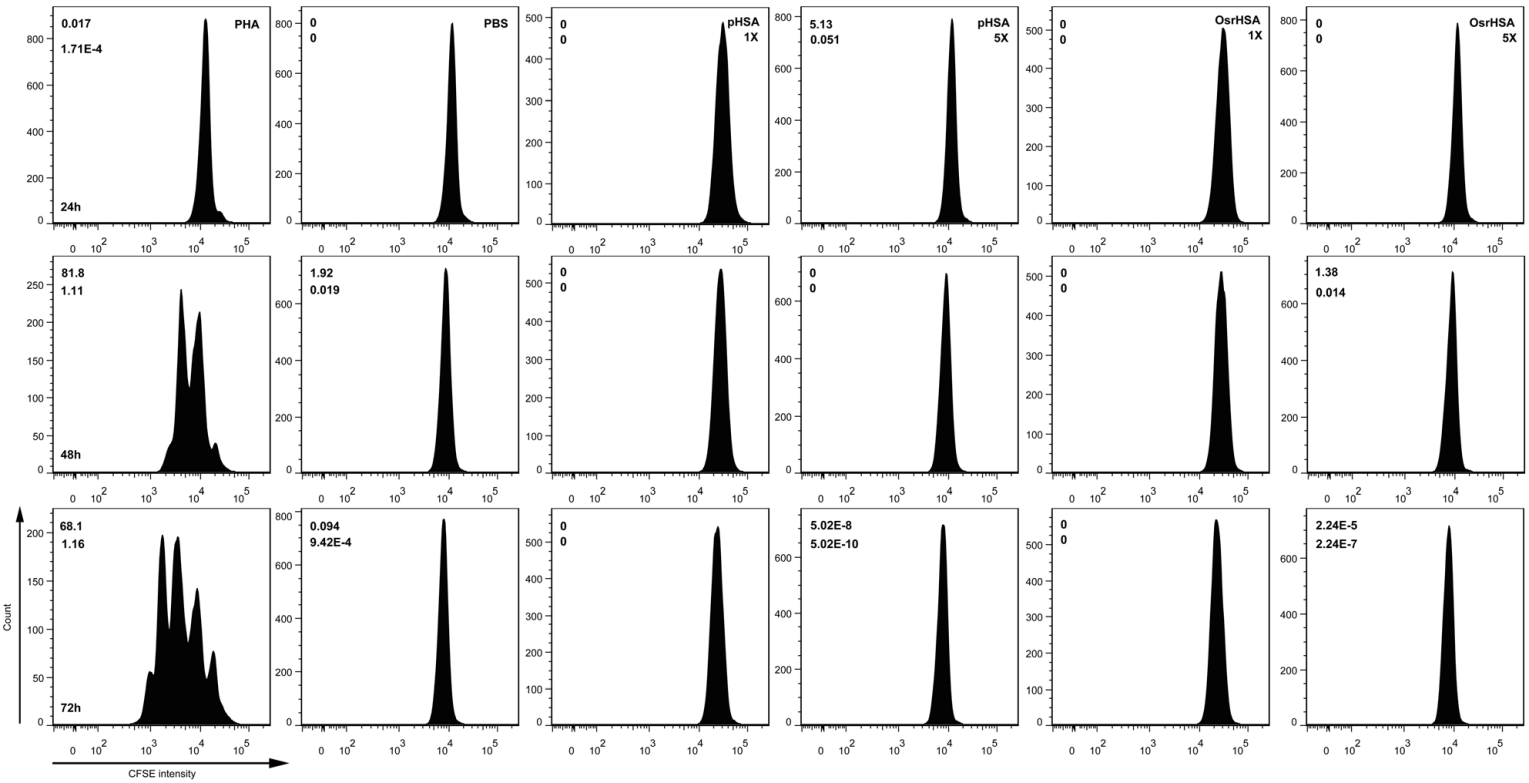
Measurement of T cell proliferation using CFSE. The PBMCs were labeled with CFSE and then treated with PHA, PBS, pHSA and OsrHSA for 24, 48 and 72

**Figure 2:**
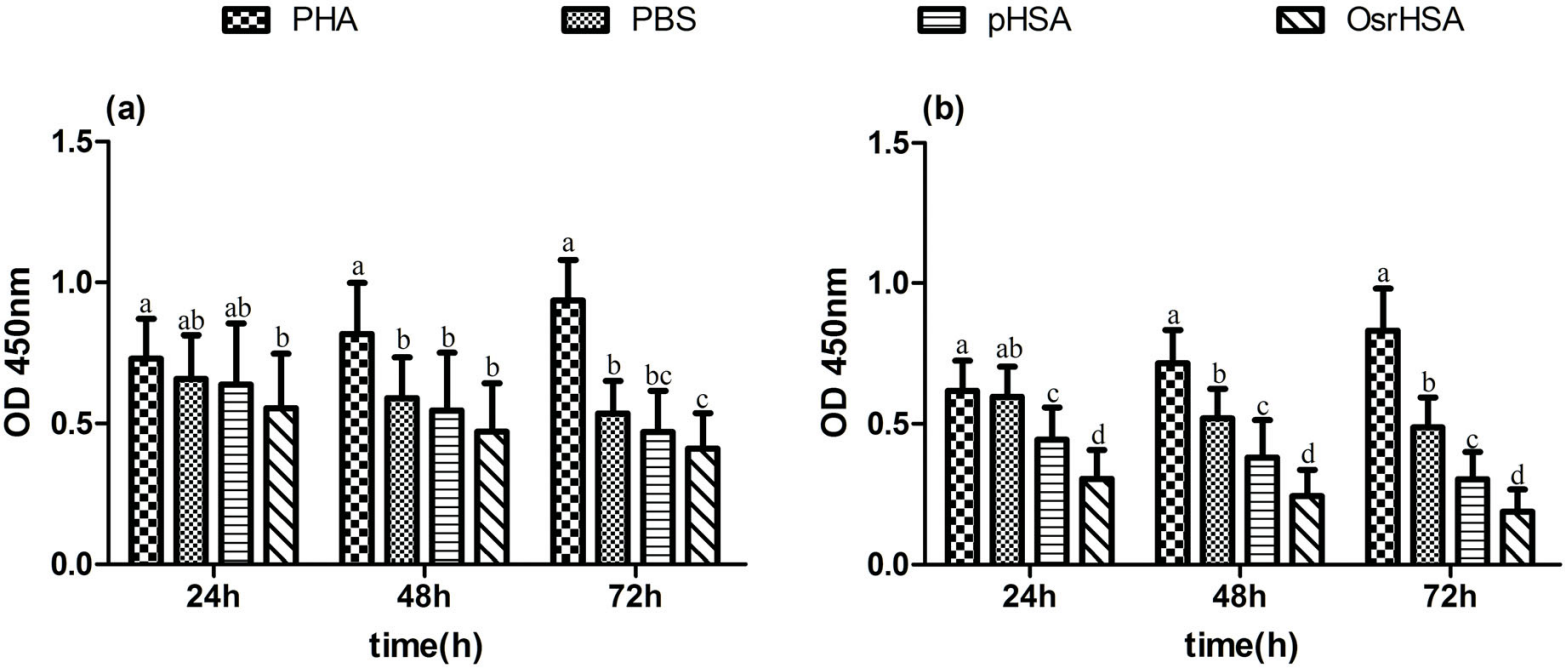
A proliferation assay was performed using a CCK-8 kit. Panel a presents the data with a 1x dosage, and panel b indicates the data with a 5x dosage. Each error bar with the same letter has the same significant level of *p* value.

T cell proliferation is an indicator of the immunogenicity of an antigen in PBMCs. When T cells encounter their cognate antigen, the naive T cells will proliferate and differentiate into helper, cytotoxic or regulatory T cells, each of which perform different immune functions [32]. Thus, lymphocyte proliferation *ex vivo* could help to assess the potential for immunotoxicity of a biopharmaceutical. To evaluate the potential immunotoxicity of the impurities in OsrHSA, a carboxyfluorescein succinimidyl ester (CFSE) cell division assay was performed using PBMCs. As shown in Fig. 1, a slow decay in CFSE fluorescence intensity over time was observed in the phytohemagglutinin (PHA) group, while cells treated with phosphate-buffered saline (PBS), pHSA or OsrHSA did not exhibit any decreases in CFSE fluorescence intensity at any time point. These results indicated that similarly to the negative control, neither OsrHSA nor pHSA stimulated T cell proliferation. To further confirm these results, we performed an alternative proliferation assay using a Cell Counting Kit-8 (CCK-8). While we observed cell proliferation over time in PHA-treated PBMCs, no cell proliferation was observed with the PBS negative control, pHSA or OsrHSA treatment. However, increased cell apoptosis was observed with OsrHSA, pHSA and PBS treatments compared with PHA treatment (Fig. 2). Additionally, abundant cell apoptosis was observed with OsrHSA treatment at a 1x dosage after 72 h compared to pHSA. Moreover, the apoptosis with OsrHSA and pHSA treatment at the 5x dosages was significantly greater than that with PBS, but a difference in apoptosis was also observed between pHSA and OsrHSA. The observed apoptosis was dosage- and time-dependent, with the extent of the apoptosis in the order: OsrHSA>pHSA>PBS>PHA. This result suggests that OsrHSA could more easily induce apoptosis in PBMCs *ex vivo*. Taken together, the results demonstrated that similarly to the negative control, neither OsrHSA nor pHSA stimulated T cell proliferation *ex vivo* in PBMC model.

### OsrHSA-treated T cells have similar subset profiles as pHSA-treated cells

A change in T cell subsets is a very important characteristic that is highly related to changes in immune functions [33]. Two types of T cell subsets, CD4^+^ helper T cells and CD8^+^ cytotoxic T cells, have been widely used to distinguish different immune phenotypes. Evaluation of these subsets has been an important criteria of assessing a drug’s effect on the immune response [32]. Helper T (CD4^+^) cells interact with antigens presented by major histocompatibility complex (MHC) class II molecules that are capable of promoting an immune response that drives B-cell mediated antibody production. However, cytotoxic T (CD8^+^) cells interact with antigens presented by MHC class I molecules, promoting an immune response that drives cytotoxicity. Both of these cell types can have a high-avidity interaction with self MHC, causing cell death. Alterations in the CD4^+^ and CD8^+^ profiles could reflect changes in the overall condition of the immune system due to immunotoxicity. To understand whether the profiles difference of CD4^+^ and CD8^+^ T cell could present between pHSA and OsrHSA, fluorescence-activated cell sorting (FACS) analysis was performed to monitor the profiles of the CD4^+^ and CD8^+^ T cell subsets. At 24 h, the percentage of CD4^+^ T cells was not significantly different between any of the treatments at 1x or 5x dosages, while the CD4^+^ T cell percentages in the PHA-treated cells were significantly lower than those in the PBS-, pHSA- and OsrHSA-treated cells at 48 h and 72 h (Table 2), with time- and dosage-dependent effects observed. The similarity in the CD4^+^ T cell percentages between the conditions suggests that there is no difference in antibody production between pHSA- and OsrHSA-treated cells. Moreover, there was no significant difference in the CD8^+^ T cell percentage or the ratio of CD4^+^/CD8^+^ T cells observed between the pHSA- and OsrHSA-treated cells. However, significant differences were noted between the PHA-treated cells and the other groups at 48 h and 72 h (Table 2). These results indicate that OsrHSA and pHSA treatment resulted in similar CD4^+^ and CD8^+^ T cell profiles and a similar ratio of CD4^+^/CD8^+^ T cells, demonstrating that OsrHSA does not have any drug-related immunotoxic effects in PBMC model.

**Table 2:** T cell immunophenotypes at different dosages and time points of treatment.

Treatments	Cell markers			Dosages	Endpoints
	CD4^+^ (%)	CD8^+^ (%)	CD4^+^/CD8^+^		
PHA	31.30±10.60	28.83±10.62	1.23±0.60		24 h
PBS	34.31±8.91	29.47±6.36	1.23±0.44		
pHSA	33.56±9.01	32.34±5.10	1.08±0.38	1x	
OsrHSA	32.80±8.28	32.37±5.92	1.06±0.40		
pHSA	34.83±8.43	27.29±6.26	1.33±0.41	5x	
OsrHSA	35.74±9.73	28.51±6.74	1.31±0.47		
PHA	23.39±9.33	25.54±10.26	1.06±0.56		48 h
PBS	34.47±10.08**	29.27±5.78	1.24±0.47		
pHSA	36.74±10.28**	32.06±4.93*^a^	1.18±0.41	1x	
OsrHSA	35.74±9.00**	33.06±4.81**	1.11±0.37		
pHSA	37.51±9.77**	27.35±5.91	1.42±0.46*	5x	
OsrHSA	38.65±9.77**	28.86±5.70	1.40±0.48*		
PHA	23.18±8.74	29.08±12.01	0.92±0.50		72 h
PBS	37.19±8.97**	29.47±6.27	1.29±0.44*		
pHSA	36.44±9.37**	33.22±5.24	1.14±0.40	1x	
OsrHSA	36.94±9.23**	33.40±5.29	1.14±0.39		
pHSA	39.76±9.52**	29.94±6.32	1.40±0.47*	5x	
OsrHSA	40.21±9.76**	30.04±6.47	1.41±0.49*		

a. *p<0.05; **p<0.01.

Comparisons for significant differences were evaluated between the PHA group and an alternative group (PBS, pHSA and OsrHSA); the PBS, pHSA and OsrHSA groups had no significant differences between each other after evaluation by *t*-test.

### OsrHSA-treated cells secrete the same level of cytokines as pHSA-treated cells

**Figure 3:**
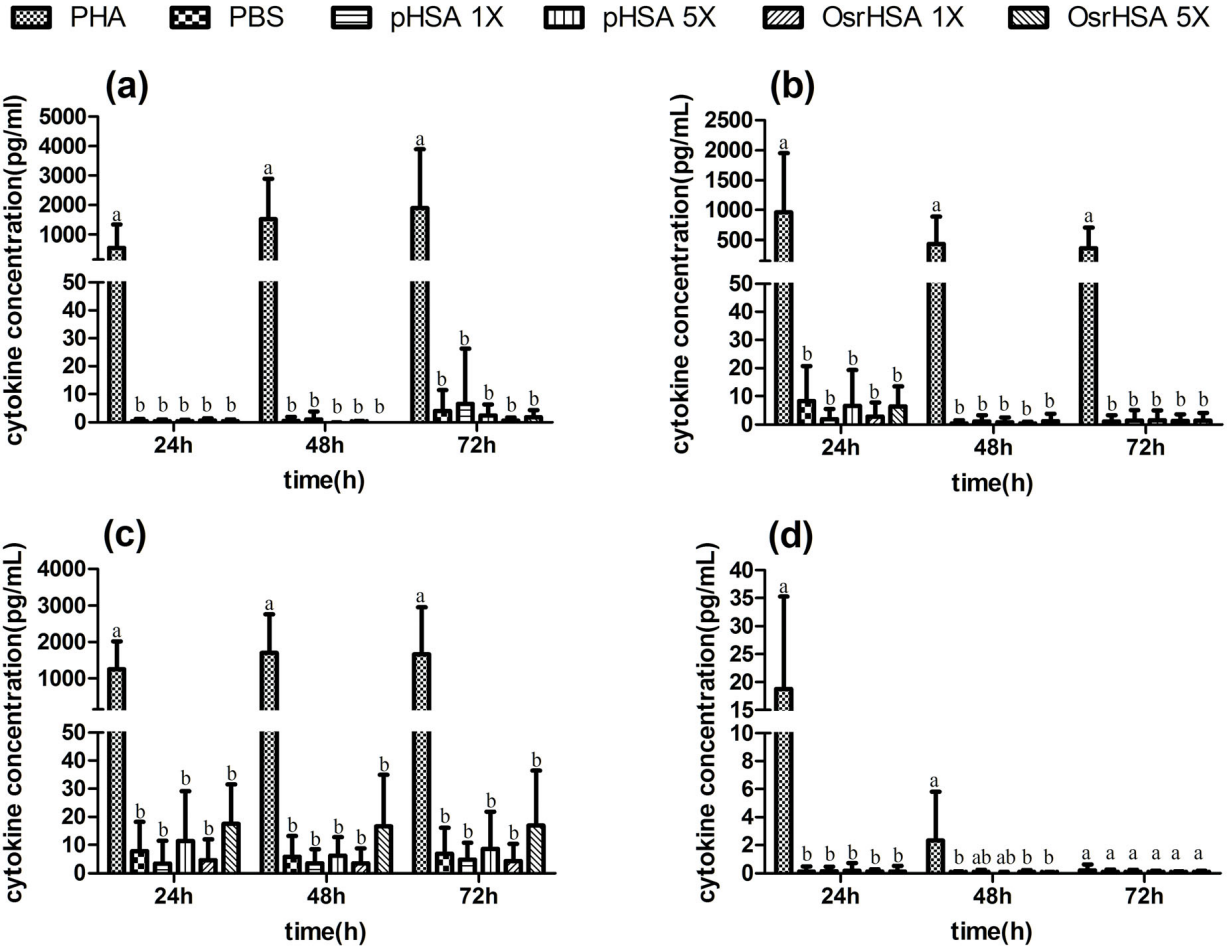
The profiles of four cytokines in PBMCs following different treatments. The levels of cytokines were assayed using a CBA kit. Panels a, b, c and d present the data for the cytokines IFN-γ, TNF-α, IL-10 and IL-4, respectively. Each error bar with the same letter has the same significant level of *p* value.

Cytokines are important cell mediators in the immune system, modulating cell growth, migration, differentiation and response to chemicals [34]. Increases in the cytokine levels reflect potential inflammation and immunotoxicity. Evaluation of cytokine release has been reported to be a promising tool to identify a potential risk for eliciting adverse pro-inflammatory reactions; thus, this approach is ideal for screening novel biopharmaceuticals [35], [36]. To predict whether OsrHSA itself or impurities in the compound could potentially induce anaphylaxis, immune suppression or inflammation in the human body, we monitored the levels of IL-4, which regulates IgE production; IL-10, which suppresses the immune response; and IFN-γ and TNF-α, which promote inflammation in PBMCs [36]. As shown in Fig. 3, the four cytokines were significantly elevated in PHA-treated cells compared to pHSA-, OsrHSA- and PBS-treated cells at different dosages and time points (Fig. 3 and Table S1, S2, S3, S4). The levels of the four cytokines in pHSA-, OsrHSA- and PBS-treated cells were all similar, and all were observed to be at the background level. To further evaluate the cytokine levels between all of the treatments, we performed hierarchical cluster analysis (HCA). The results showed that the OsrHSA-, pHSA- and PBS-treated cells were clustered in the same category and that the PHA-treated cells belonged in another (Fig. S1). These results indicate that OsrHSA has the same effect as pHSA and PBS on cytokine levels *ex vivo*. Thus, our results demonstrate that OsrHSA has the same immunotoxicity as pHSA in PBMC model.

## Discussion

HSA is a carrier protein that stabilizes the extracellular fluid volume [24] and is extensively used clinically to treat hypoproteinemia, hemorrhagic shock, serious burn injuries, cirrhotic ascites and fetal erythroblastosis [37], [38]. The dosage of HSA is at least 10 g per 60 kg body weight for clinical applications, which indicates that the human body would be exposed to a large amount of impurities at a megadosage, even if the purity of recombinant HSA were as high as 99.99%. The potential for the risk of immunoreaction to the impurities arises, especially in a new expression system that would be used for clinical applications. To completely eliminate the possibility of a “cytokine storm” similar to what occurred in TGN1412, it is essential to assess the risk in a preclinical safety evaluation when the products from a new expression system will first be used for clinical applications. In the present study, we used human PBMCs to evaluate the *ex vivo* immunotoxicity potential of OsrHSA. The data indicate that OsrHSA has similar *ex vivo* effects as pHSA on T lymphocyte proliferation, T cell immunophenotyping and cytokine production, demonstrating that OsrHSA is as safe as pHSA. Furthermore, we have completed immunotoxicity tests of OsrHSA using rat or cynomolgus monkey models following the guideline of China Food and Drug Administration (CFDA). The data indicated that no differences in inflammation, immunogenicity and immunotoxicity were observed between OsrHSA and pHSA. The PBMC model can be used as a complementary method to predict potential immunotoxicity in further clinical trials with a novel expression system.

Recently, protein O-linked glycosylation of HSA has been identified as a significant cause for concern with respect to inducing an immune response in humans. The biosynthesis of O-linked sugar chains is initiated in the ER and catalyzed by mannosyltransferase by the addition of GalNAc from UDP-GalNAc to threonine or serine residues in the polypeptide [39]. The consensus sequence for the O-glycosylation of mucin-type peptide contains the pentapeptide, XTPXP, XTXXP, and XTPXX [40]. Several genes responding for O-linked glycosylation has been identified in yeast (*Saccharomyces Cerevisiae*) [41], [42]. To reduce of O-linked glycosylation, an engineered yeast strain deficient in protein mannosyltransferases effective at controlling O-linked glycosylation has been developed [43]. Moreover, a mucin-type O-linked glycosylation has been reported in basic glutelin in rice endosperm [44]. Therefore, the potential of O-linked glycosylation could occur when foreign protein is expressed in rice endosperm. To determine if there is any O-linked glycosylation in OsrHSA, we first checked the sequence of HSA. Two consensus sequences KTPVS (465–470) and ETYVP (495–500) for the O-glycosylation of mucin-type were found. Next, we analyzed the data of full-length protein sequencing of OsrHSA, O-linked glycosylated forms of rice derived recombinant HSA were not detected using MALDI-TOF/TOF.

A significant and dose-dependent difference in apoptosis was observed between PBS- and OsrHSA/pHSA-treated cells. In recent years, several pieces of evidence have demonstrated that albumin (HSA or BSA) overloading can induce apoptosis by causing endoplasmic reticulum stress in a time- and dose-dependent manner [45], [46]. Hence, the increased apoptosis in OsrHSA- or pHSA-treated cells compared to PBS-treated cells could be due to HSA overloading. It is also possible that HSA could change the osmotic pressure *ex vivo* to promote cell apoptosis. Furthermore, it is possible that the increased number of CD4^+^ T cells could interact with CD8^+^ T cells to promote PBMC apoptosis via a high-avidity interaction with self MHC. Regarding the increased T cell apoptosis observed in OsrHSA-treated cells compared to pHSA-treated cells, several the possible hypotheses could explain the increased apoptosis in OsrHSA. Our previous data has showed there is a difference in the lipid types between OsrHSA and pHSA [6]. Eight types of lipids present in pHSA have been absent in OsrHSA. Studies indicated that several of those lipids might be involved in T cell proliferation and apoptosis [47], [48], [49], [50], [51], [52]. Thus, the absence of those lipids in OsrHSA may account for the difference in apoptosis. Furthermore, there are approximately 4% impurities in pHSA (purity 96%), suggesting that pHSA might contain some unknown factors that could impede T cell apoptosis in *ex vivo* PBMC model.

Various *in vitro* or *ex vivo* approaches could be used to evaluate the risk potential of biopharmaceuticals, such as mAbs, chemicals or xenobiotics [36], [53], [54]. The FDA encourages using an alternative *ex vivo* system to predict the potential for immunotoxicity as a complement to a standard nonclinical trial. In the present study, we used PBMCs to assess OsrHSA produced in rice endosperm cells, which will be used in large doses for clinical applications. Our results indicated that OsrHSA, as a novel PMP product, is safe as pHSA, demonstrating its minimal immunotoxicity before it goes to an FIH trial. Our results also suggest that because rice is a staple food, humans may have acquired a high tolerance for rice antigens during evolution. We also provide indirect evidence that rice could be a favored bioreactor for PMP production. Our data provide an example of using human immune cells *ex vivo* to predict the risk of immunotoxicity of a PMP product. This *ex vivo* system also provides an alternative approach for predicting the potential risks of novel biopharmaceuticals before clinical trial, which could be a valuable complement to immune toxicity assessment.

## Conclusions

In summary, we assessed the potential for immunotoxicity of OsrHSA by monitoring T cell proliferation, the profiles of CD4^+^ and CD8^+^ T cells and the levels of four cytokines using an *ex vivo* PBMC model. Our data showed that OsrHSA, similarly to pHSA, did not activate T cells, change the profiles of CD4^+^ and CD8^+^ T cells or elevate the cytokine levels. Our results demonstrated that OsrHSA has equivalent immunotoxicity to pHSA in PBMC model.

## Supporting Information

Figure S1 **Hierarchical cluster analysis of the four cytokine profiles for cells treated with PHA, PBS, pHSA and OsrHSA.**
(TIF)

Table S1 **Individual result of IFN-γ production.**
(DOC)

Table S2 **Individual result of TNF-α production.**
(DOCX)

Table S3 **Individual result of IL-10 production.**
(DOCX)

Table S4 **Individual result of IL-4 production.**
(DOCX)

## References

[1] Fischer R, Schillberg S, Hellwig S, Twyman RM, Drossard J (2012) GMP issues for recombinant plant-derived pharmaceutical proteins. Biotechnol Adv 30: 434–439. 21856403 10.1016/j.biotechadv.2011.08.007

[2] Ou J, Guo Z, Shi J, Wang X, Liu J, et al (2014) Transgenic rice endosperm as a bioreactor for molecular pharming. Plant Cell Reports 33: 585–594. 24413763 10.1007/s00299-013-1559-2

[3] Obembe OO, Popoola JO, Leelavathi S, Reddy SV (2011) Advances in plant molecular farming. Biotechnol Adv 29: 210–222. 21115109 10.1016/j.biotechadv.2010.11.004

[4] Oszvald M, Kang TJ, Tomoskozi S, Jenes B, Kim TG, et al (2008) Expression of cholera toxin B subunit in transgenic rice endosperm. Mol Biotechnol 40: 261–268. 18618297 10.1007/s12033-008-9083-2

[5] Zhang D, Nandi S, Bryan P, Pettit S, Nguyen D, et al (2010) Expression, purification, and characterization of recombinant human transferrin from rice (Oryza sativa L.). Protein Expr Purif 74: 69–79. 20447458 10.1016/j.pep.2010.04.019 PMC2926268

[6] He Y, Ning T, Xie T, Qiu Q, Zhang L, et al (2011) Large-scale production of functional human serum albumin from transgenic rice seeds. Proc Natl Acad Sci U S A 108: 19078–19083. 22042856 10.1073/pnas.1109736108 PMC3223471

[7] Zhang L, Shi J, Jiang D, Stupak J, Ou J, et al (2012) Expression and characterization of recombinant human alpha-antitrypsin in transgenic rice seed. J Biotechnol 164: 300–308. 23376844 10.1016/j.jbiotec.2013.01.008

[8] An N, Ou J, Jiang D, Zhang L, Liu J, et al (2013) Expression of a functional recombinant human basic fibroblast growth factor from transgenic rice seeds. Int J Mol Sci 14: 3556–3567. 23434658 10.3390/ijms14023556 PMC3588058

[9] Latta M, Knapp M, Sarmientos P, Bréfort G, Becquart J, et al (1987) Synthesis and purification of mature human serum albumin from E. coli. Nature Biotechnology 5: 1309–1314.

[10] Sleep D, Belfield G, Goodey A (1990) The secretion of human serum albumin from the yeast Saccharomyces cerevisiae using five different leader sequences. Biotechnology 8: 42–46. 1366511 10.1038/nbt0190-42

[11] Fleer R, Yeh P, Amellal N, Maury I, Fournier A, et al (1991) Stable Multicopy Vectors for High–Level Secretion of Recombinant Human Serum Albumin by Kluyveromyces Yeasts. Nature Biotechnology 9: 968–975. 10.1038/nbt1091-968 1367806

[12] Kobayashi K, Kuwae S, Ohya T, Ohda T, Ohyama M, et al (2000) High-level expression of recombinant human serum albumin from the methylotrophic yeast Pichia pastoris with minimal protease production and activation. Journal of Bioscience and Bioengineering 89: 55–61. 16232698 10.1016/s1389-1723(00)88050-0

[13] Barash I, Faerman A, Baruch A, Nathan M, Hurwitz DR, et al (1993) Synthesis and secretion of human serum albumin by mammary gland explants of virgin and lactating transgenic mice. Transgenic Res 2: 266–276. 8242096 10.1007/BF01968839

[14] Farran I, Sanchez-Serrano JJ, Medina JF, Prieto J, Mingo-Castel AM (2002) Targeted expression of human serum albumin to potato tubers. Transgenic Res 11: 337–346. 12212837 10.1023/a:1016356510770

[15] Fernandez-San Millan A, Mingo-Castel A, Miller M, Daniell H (2003) A chloroplast transgenic approach to hyper-express and purify Human Serum Albumin, a protein highly susceptible to proteolytic degradation. Plant Biotechnol J 1: 71–79. 17147744 10.1046/j.1467-7652.2003.00008.x PMC3481847

[16] Huang LF, Liu YK, Lu CA, Hsieh SL, Yu SM (2005) Production of human serum albumin by sugar starvation induced promoter and rice cell culture. Transgenic Res 14: 569–581. 16245148 10.1007/s11248-004-6481-5

[17] Sijmons PC, Dekker BMM, Schrammeijer B, Verwoerd TC, Van Den Elzen PJM, et al (1990) Production of correctly processed human serum albumin in transgenic plants. Nature Biotechnology 8: 217–221. 10.1038/nbt0390-217 1366404

[18] Kasahara A, Kita K, Tomita E, Toyota J, Imai Y, et al (2008) Repeated administration of recombinant human serum albumin caused no serious allergic reactions in patients with liver cirrhosis: a multicenter clinical study. Journal of gastroenterology 43: 464–472. 18600391 10.1007/s00535-008-2178-5

[19] Schindel F, Andresen C, Bosse D (2003) Comparison of Recombinant Human Albumin with Human Serum Albumin: Safety, Tolerability and Pharmacodynamics. J Clinical Pharmacology 43: 1032. 10.1177/0091270004269646 15601806

[20] Cavagnaro JA (2008) Preclinical safety evaluation of biopharmaceuticals: a science-based approach to facilitating clinical trials: John Wiley & Sons.

[21] Frantz S (2007) Pharma faces major challenges after a year of failures and heated battles. Nat Rev Drug Discov 6: 5–7. 17269157 10.1038/nrd2230

[22] Suntharalingam G, Perry MR, Ward S, Brett SJ, Castello-Cortes A, et al (2006) Cytokine storm in a phase 1 trial of the anti-CD28 monoclonal antibody TGN1412. N Engl J Med 355: 1018–1028. 16908486 10.1056/NEJMoa063842

[23] Stebbings R, Findlay L, Edwards C, Eastwood D, Bird C, et al (2007) “Cytokine storm” in the phase I trial of monoclonal antibody TGN1412: better understanding the causes to improve preclinical testing of immunotherapeutics. J Immunol 179: 3325–3331. 17709549 10.4049/jimmunol.179.5.3325

[24] Delves PJ, Martin SJ, Burton DR, Roitt IM (2011) Roitt’s essential immunology: John Wiley & Sons.

[25] Stebbings R, Poole S, Thorpe R (2009) Safety of biologics, lessons learnt from TGN1412. Curr Opin Biotechnol 20: 673–677. 19892543 10.1016/j.copbio.2009.10.002

[26] Joubert MK, Hokom M, Eakin C, Zhou L, Deshpande M, et al (2012) Highly aggregated antibody therapeutics can enhance the in vitro innate and late-stage T-cell immune responses. J Biol Chem 287: 25266–25279. 22584577 10.1074/jbc.M111.330902 PMC3408134

[27] Ogwang C, Afolabi M, Kimani D, Jagne YJ, Sheehy SH, et al (2013) Safety and immunogenicity of heterologous prime-boost immunisation with Plasmodium falciparum malaria candidate vaccines, ChAd63 ME-TRAP and MVA ME-TRAP, in healthy Gambian and Kenyan adults. PLoS One 8: e57726. 23526949 10.1371/journal.pone.0057726 PMC3602521

[28] Olsen SC, Johnson C (2012) Immune responses and safety after dart or booster vaccination of bison with Brucella abortus strain RB51. Clin Vaccine Immunol 19: 642–648. 22461528 10.1128/CVI.00033-12 PMC3346322

[29] Whelan KT, Pathan AA, Sander CR, Fletcher HA, Poulton I, et al (2009) Safety and immunogenicity of boosting BCG vaccinated subjects with BCG: comparison with boosting with a new TB vaccine, MVA85A. PLoS One 4: e5934. 19529780 10.1371/journal.pone.0005934 PMC2694271

[30] Finco D, Grimaldi C, Fort M, Walker M, Kiessling A, et al (2014) Cytokine release assays: Current practices and future directions. Cytokine 66: 143–155. 24412476 10.1016/j.cyto.2013.12.009

[31] Walker MR, Makropoulos DA, Achuthanandam R, Van Arsdell S, Bugelski PJ (2011) Development of a human whole blood assay for prediction of cytokine release similar to anti-CD28 superagonists using multiplex cytokine and hierarchical cluster analysis. International immunopharmacology 11: 1697–1705. 21689786 10.1016/j.intimp.2011.06.001

[32] Herzyk DJ, Bussiere JL (2008) Immunotoxicology strategies for pharmaceutical safety assessment: John Wiley & Sons.

[33] Baroncelli S, Panzini G, Geraci A, Pardini S, Corrias F, et al (1997) Longitudinal characterization of CD4, CD8 T-cell subsets and of haematological parameters in healthy newborns of cynomolgus monkeys. Vet Immunol Immunopathol 59: 141–150. 9437832 10.1016/s0165-2427(97)00040-8

[34] Foster JR (2001) The functions of cytokines and their uses in toxicology. Int J Exp Pathol 82: 171–192. 11488991 10.1046/j.1365-2613.2001.iep0082-0171-x PMC2517710

[35] House RV (1999) Theory and practice of cytokine assessment in immunotoxicology. Methods 19: 17–27. 10525434 10.1006/meth.1999.0823

[36] Stolevik SB, Nygaard UC, Namork E, Granum B, Pellerud A, et al (2011) In vitro cytokine release from human peripheral blood mononuclear cells in the assessment of the immunotoxic potential of chemicals. Toxicol In Vitro 25: 555–562. 21144890 10.1016/j.tiv.2010.11.021

[37] Alexander MR, Ambre JJ, Liskow BI, Trost DC (1979) Therapeutic use of albumin. JAMA 241: 2527–2529. 439338

[38] Hastings GE, Wolf PG (1992) The therapeutic use of albumin. Archives of Family Medicine 1: 281–287. 1341604 10.1001/archfami.1.2.281

[39] Wu AM, Csako G, Herp A (1994) Structure, biosynthesis, and function of salivary mucins. Mol Cell Biochem 137: 39–55. 7845377 10.1007/BF00926038

[40] Yoshida A, Suzuki M, Ikenaga H, Takeuchi M (1997) Discovery of the shortest sequence motif for high level mucin-type O-glycosylation. J Biol Chem 272: 16884–16888. 9201996 10.1074/jbc.272.27.16884

[41] Lussier M, Gentzsch M, Sdicu A-M, Bussey H, Tanner W (1995) Protein O-Glycosylation in Yeast the PMT2 gene specifies a second protein O-Maanosyltransferase that functions in addition to the PMT1-encoded activity. Journal of Biological Chemistry 270: 2770–2775. 7852348 10.1074/jbc.270.6.2770

[42] Strahl-Bolsinger S, Immervoll T, Deutzmann R, Tanner W (1993) PMT1, the gene for a key enzyme of protein O-glycosylation in Saccharomyces cerevisiae. Proc Natl Acad Sci U S A 90: 8164–8168. 8367478 10.1073/pnas.90.17.8164 PMC47309

[43] Bobrowicz P, Cook WJ, Kett W (2012) Production of glycoproteins with reduced O-glycosylation. Google Patents.

[44] Kishimoto T, Watanabe M, Mitsui T, Hori H (1999) Glutelin Basic Subunits Have a Mammalian Mucin-Type O-Linked Disaccharide Side Chain. Archives of Biochemistry and Biophysics 370: 271–277. 10510286 10.1006/abbi.1999.1406

[45] Czyz CN, Foster JA, Lam VB, Holck DE, Wulc AE, et al (2012) Efficacy of pulsed electromagnetic energy in postoperative recovery from blepharoplasty. Dermatol Surg 38: 445–450. 22092688 10.1111/j.1524-4725.2011.02215.x

[46] Okamura K, Dummer P, Kopp J, Qiu L, Levi M, et al (2013) Endocytosis of albumin by podocytes elicits an inflammatory response and induces apoptotic cell death. PLoS One 8: e54817. 23382978 10.1371/journal.pone.0054817 PMC3557279

[47] Moolenaar WH (1991) Mitogenic action of lysophosphatidic acid. Adv Cancer Res 57: 87–102. 1950708 10.1016/s0065-230x(08)60996-3

[48] Moolenaar WH, van Corven EJ (1990) Growth factor-like action of lysophosphatidic acid: mitogenic signalling mediated by G proteins. Ciba Found Symp 150: 99–106 discussion 106–111. 2115427 10.1002/9780470513927.ch7

[49] Tigyi G, Dyer DL, Miledi R (1994) Lysophosphatidic acid possesses dual action in cell proliferation. Proc Natl Acad Sci U S A 91: 1908–1912. 8127904 10.1073/pnas.91.5.1908 PMC43273

[50] Tomita M, Baker RC, Ando S, Santoro TJ (2004) Arachidonoyl-phospholipid remodeling in proliferating murine T cells. Lipids Health Dis 3: 1. 14754461 10.1186/1476-511X-3-1 PMC343295

[51] Wongtangtintharn S, Oku H, Iwasaki H, Inafuku M, Toda T, et al (2005) Incorporation of branched-chain fatty acid into cellular lipids and caspase-independent apoptosis in human breast cancer cell line, SKBR-3. Lipids in health and disease 4: 1476–1511X. 10.1186/1476-511X-4-29 PMC1315321 16305741

[52] Takahashi M, Okazaki H, Ogata Y, Takeuchi K, Ikeda U, et al (2002) Lysophosphatidylcholine induces apoptosis in human endothelial cells through a p38-mitogen-activated protein kinase-dependent mechanism. Atherosclerosis 161: 387–394. 11888522 10.1016/s0021-9150(01)00674-8

[53] Kooijman R, Devos S, Hooghe-Peters E (2010) Inhibition of in vitro cytokine production by human peripheral blood mononuclear cells treated with xenobiotics: implications for the prediction of general toxicity and immunotoxicity. Toxicol In Vitro 24: 1782–1789. 20633635 10.1016/j.tiv.2010.07.007

[54] Vidal JM, Kawabata TT, Thorpe R, Silva-Lima B, Cederbrant K, et al (2010) In vitro cytokine release assays for predicting cytokine release syndrome: the current state-of-the-science. Report of a European Medicines Agency Workshop. Cytokine 51: 213–215. 20471854 10.1016/j.cyto.2010.04.008

