# Supplementary figures and images for: Immunotoxicity Assessment of Rice-Derived Recombinant Human Serum Albumin Using Human Peripheral Blood Mononuclear Cells
Source: PLoS One. 2014 Aug 6;9(8):e104426. doi: 10.1371/journal.pone.0104426 (PMC4123919; doi:10.1371/journal.pone.0104426)

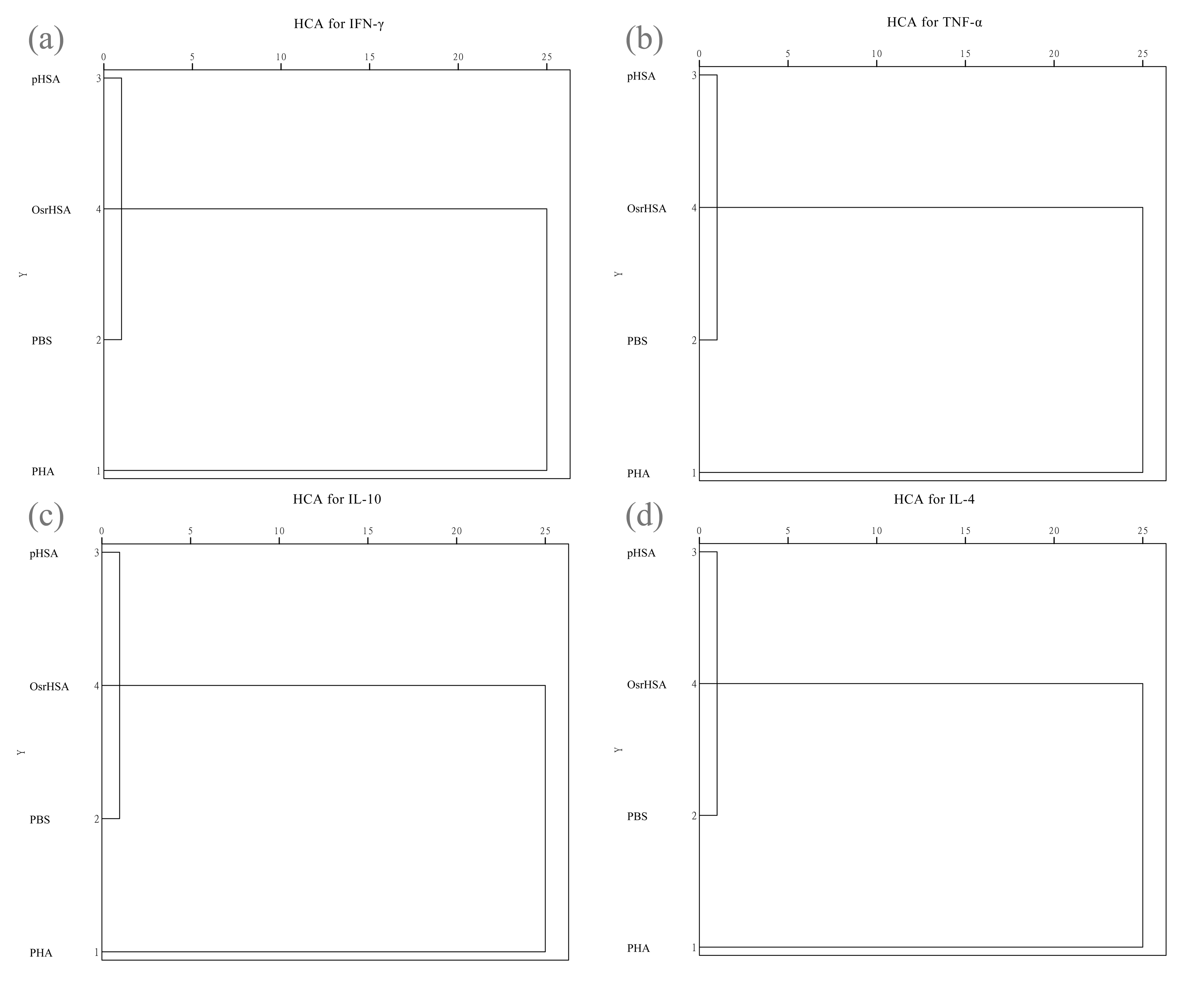

Supplement: Figure S1 — Hierarchical cluster analysis of the four cytokine profiles for cells treated with PHA, PBS, pHSA and OsrHSA. (TIF) [file pone.0104426.s001.tif]
