# Supplementary material for: Immunotoxicity Assessment of Rice-Derived Recombinant Human Serum Albumin Using Human Peripheral Blood Mononuclear Cells
Source: PLoS One. 2014 Aug 6;9(8):e104426. doi: 10.1371/journal.pone.0104426 (PMC4123919; doi:10.1371/journal.pone.0104426)
Supplement: Table S1 — Individual result of IFN-γ production. (DOC) [file pone.0104426.s002.doc]

**Table S1.** Individual result of IFN-γ production

| **Donor No.** | **Gender** | **PHA** | | | **PBS** | | | **pHSA** | | | **OsrHSA** | | |
| --- | --- | --- | --- | --- | --- | --- | --- | --- | --- | --- | --- | --- | --- |
|  |  | **24h** | **48h** | **72h** | **24h** | **48h** | **72h** | **24h** | **48h** | **72h** | **24h** | **48h** | **72h** |
| 1 | Male | 894.825 | 1608.17 | 1892.775 | 2.725 | 1.665 | 1.775 | 2.04 | 0 | 0 | 1.58 | 0.005 | 0.345 |
| 2 | Male | 121.995 | 477.41 | 388.195 | 0 | 0.93 | 0 | 0 | 1.15 | 0 | 0.12 | 0.865 | 0 |
| 3 | Male | 94.195 | 1125.76 | 2198.92 | 0 | 0 | 0 | 0 | 0 | 0 | 0 | 0 | 0 |
| 4 | Male | 123.995 | 437.3 | 221.645 | 0 | 0 | 2.445 | 0 | 0 | 0 | 0 | 0 | 3.745 |
| 5 | Male | 55.38 | 197.285 | 464.005 | 0 | 0 | 0 | 0 | 0 | 0 | 0 | 0 | 0 |
| 6 | Male | 178.275 | 756.74 | 794.995 | 0 | 6.2 | 9.545 | 0 | 0 | 0 | 0 | 0 | 5.805 |
| 7 | Male | 1165.55 | 3990.91 | 5455.96 | 0 | 0 | 7.325 | 0 | 0 | 5.185 | 0 | 0 | 0.16 |
| 8 | Male | 1200.505 | 4572.91 | 4425.87 | 0 | 0 | 0.215 | 0 | 0 | 0 | 0 | 0 | 2.63 |
| 9 | Male | 230.25 | 992.51 | 765.95 | 0 | 0 | 0.245 | 0 | 0 | 2.13 | 0 | 0 | 0 |
| 10 | Male | 227.205 | 230.875 | 253.735 | 0 | 0 | 10.445 | 0 | 0 | 0 | 0 | 0 | 0 |
| 11 | Female | 3504.15 | 1357.89 | 5179.77 | 0 | 0 | 0 | 0 | 0 | 0 | 0 | 0 | 0 |
| 12 | Female | 191.965 | 707.42 | 1260.675 | 1.24 | 0.95 | 1.235 | 1.085 | 0.87 | 3.115 | 1.085 | 0.835 | 1.12 |
| 13 | Female | 532.03 | 1542.44 | 1894.625 | 0 | 0 | 0 | 0 | 0 | 0.095 | 2.3 | 0 | 0.6 |
| 14 | Female | 370.545 | 3732.775 | 6993.025 | 0 | 0 | 0 | 0 | 0 | 0 | 0 | 0 | 0 |
| 15 | Female | 928.84 | 3372.18 | 2140.695 | 0.865 | 0 | 0 | 0 | 8.78 | 62.63 | 0.835 | 0 | 0 |
| 16 | Female | 361.035 | 2556.81 | 1140.745 | 1.94 | 0 | 0 | 2.09 | 0.09 | 0 | 2.505 | 0 | 0 |
| 17 | Female | 337.6 | 925.19 | 980.715 | 0 | 0 | 27 | 0 | 0 | 5.915 | 0 | 0 | 3.35 |
| 18 | Female | 84 | 301.005 | 103.505 | 1.55 | 0.865 | 19.47 | 0.71 | 0 | 11.405 | 0 | 0 | 6.4 |
| 19 | Female | 328.71 | 1249.72 | 1017.92 | 0 | 0 | 0 | 0 | 0 | 0 | 0 | 0 | 0 |
| 20 | Female | 81.23 | 382.24 | 322.695 | 0 | 0.095 | 0.095 | 0 | 0 | 0 | 0 | 0 | 0.295 |
