# Supplementary material for: Immunotoxicity Assessment of Rice-Derived Recombinant Human Serum Albumin Using Human Peripheral Blood Mononuclear Cells
Source: PLoS One. 2014 Aug 6;9(8):e104426. doi: 10.1371/journal.pone.0104426 (PMC4123919; doi:10.1371/journal.pone.0104426)
Supplement: Table S2 — Individual result of TNF-α production. (DOCX) [file pone.0104426.s003.docx]

**Table S2.** Individual result of TNF-α production

| **Donor No.** | **Gender** | **PHA** | | | **PBS** | | | **pHSA** | | | **OsrHSA** | | |
| --- | --- | --- | --- | --- | --- | --- | --- | --- | --- | --- | --- | --- | --- |
|  |  | **24h** | **48h** | **72h** | **24h** | **48h** | **72h** | **24h** | **48h** | **72h** | **24h** | **48h** | **72h** |
| 1 | Male | 1774.4 | 520.68 | 378.695 | 23.995 | 0 | 7.3 | 7.875 | 0.63 | 0.22 | 2.91 | 0.17 | 4.26 |
| 2 | Male | 267.24 | 191.955 | 116.625 | 0 | 1.195 | 0 | 0 | 1.28 | 0 | 0.205 | 1.135 | 0 |
| 3 | Male | 802.56 | 559.61 | 382.465 | 0 | 0 | 0 | 0 | 0 | 0 | 0 | 0 | 0 |
| 4 | Male | 192.77 | 99.65 | 59.725 | 0 | 0 | 1.76 | 0 | 0 | 0 | 0 | 0 | 6.65 |
| 5 | Male | 179.97 | 60.02 | 61.865 | 0 | 0 | 0 | 0 | 0 | 0 | 0 | 0 | 0 |
| 6 | Male | 358.625 | 155.385 | 133.56 | 5.96 | 0 | 0.005 | 0 | 0 | 0 | 4.255 | 0 | 0 |
| 7 | Male | 1477.51 | 925.155 | 826.4 | 9.01 | 0 | 1.225 | 0.94 | 0 | 2.005 | 3.08 | 0 | 0.475 |
| 8 | Male | 2354.91 | 2007.08 | 1353.01 | 18.375 | 0 | 0 | 15.285 | 0.085 | 0 | 20.23 | 0 | 0.47 |
| 9 | Male | 261.015 | 198.63 | 176.625 | 0 | 0.195 | 0.3 | 0 | 0 | 0.855 | 0 | 0 | 0 |
| 10 | Male | 492.065 | 140.04 | 138.48 | 0 | 0 | 0 | 0 | 0 | 0 | 0 | 0 | 0 |
| 11 | Female | 4233.05 | 225.37 | 760.92 | 3.6 | 0 | 1.35 | 0 | 7.26 | 0.26 | 0 | 0 | 0 |
| 12 | Female | 753.4 | 282.145 | 260.195 | 13.58 | 2.5 | 2.555 | 9.55 | 1.595 | 1.805 | 8.585 | 1.95 | 1.655 |
| 13 | Female | 1266.855 | 623.39 | 523.44 | 0 | 0 | 0 | 0 | 0 | 0 | 15.17 | 0 | 0.415 |
| 14 | Female | 835.395 | 692.47 | 758.285 | 2.935 | 0 | 0 | 1.57 | 0 | 0 | 1.32 | 0 | 0.115 |
| 15 | Female | 1200.53 | 606.885 | 389.7 | 0 | 0 | 0 | 0 | 0.23 | 12.095 | 0 | 0 | 0 |
| 16 | Female | 411.54 | 385.715 | 198.37 | 33.69 | 0.18 | 0 | 2.25 | 0.995 | 0 | 2.105 | 0.165 | 0 |
| 17 | Female | 352.495 | 141.53 | 150.28 | 8.07 | 0 | 1.21 | 39.975 | 0 | 0.645 | 7.555 | 7.805 | 7.485 |
| 18 | Female | 42.355 | 23.96 | 10.305 | 1.315 | 4.33 | 7.235 | 7.735 | 2.73 | 11.345 | 0.365 | 3.815 | 5.225 |
| 19 | Female | 1448.38 | 714.91 | 435.62 | 42.96 | 0 | 0 | 0 | 0 | 0 | 12.96 | 0 | 0 |
| 20 | Female | 488.94 | 174.805 | 161.865 | 3.395 | 0 | 0.01 | 0 | 4.9 | 0 | 14.015 | 0 | 0 |
