# Supplementary material for: Immunotoxicity Assessment of Rice-Derived Recombinant Human Serum Albumin Using Human Peripheral Blood Mononuclear Cells
Source: PLoS One. 2014 Aug 6;9(8):e104426. doi: 10.1371/journal.pone.0104426 (PMC4123919; doi:10.1371/journal.pone.0104426)
Supplement: Table S3 — Individual result of IL-10 production. (DOCX) [file pone.0104426.s004.docx]

**Table S3.** Individual result of IL-10 production

| **Donor No.** | **Gender** | **PHA** | | | **PBS** | | | **pHSA** | | | **OsrHSA** | | |
| --- | --- | --- | --- | --- | --- | --- | --- | --- | --- | --- | --- | --- | --- |
|  |  | **24h** | **48h** | **72h** | **24h** | **48h** | **72h** | **24h** | **48h** | **72h** | **24h** | **48h** | **72h** |
| 1 | Male | 1102.47 | 1193.72 | 827.965 | 7.06 | 0 | 0 | 0.995 | 0 | 0 | 2.545 | 0 | 0 |
| 2 | Male | 715.055 | 954.36 | 667.795 | 0 | 1.26 | 0.26 | 0.16 | 1.43 | 1.07 | 1.555 | 2.835 | 1.965 |
| 3 | Male | 374.51 | 605.905 | 793.19 | 0 | 0.84 | 0 | 0.02 | 0.46 | 0 | 0.28 | 0.865 | 1.095 |
| 4 | Male | 404.53 | 982.33 | 758.76 | 0.13 | 1.67 | 5.205 | 0.67 | 2.25 | 8.695 | 1.88 | 1.825 | 4.895 |
| 5 | Male | 398.555 | 513.525 | 505.41 | 0.545 | 0.955 | 2.06 | 0.98 | 0.955 | 1.87 | 1.96 | 1.425 | 2.965 |
| 6 | Male | 1306.89 | 1669.855 | 1524.69 | 11.92 | 6.71 | 7.89 | 4.485 | 2.49 | 4.785 | 13.23 | 6.9 | 7.76 |
| 7 | Male | 1823.175 | 3203.135 | 4799.43 | 5.065 | 2.055 | 6.37 | 2.025 | 0.8 | 3.535 | 5.215 | 2.66 | 7.2 |
| 8 | Male | 2342.31 | 3300.24 | 3514.78 | 11.365 | 6.01 | 8.435 | 9.555 | 6.91 | 6.69 | 24.045 | 10.535 | 15.295 |
| 9 | Male | 834.78 | 2178.37 | 2199.09 | 1.33 | 6.895 | 9.1 | 3.695 | 6.81 | 7.265 | 5.15 | 18.43 | 11.5 |
| 10 | Male | 2894.195 | 2845.73 | 3481.45 | 13.55 | 19.455 | 24.05 | 14.165 | 12.755 | 12.425 | 24.775 | 23.215 | 22.495 |
| 11 | Female | 898.77 | 126.27 | 382.61 | 0 | 0 | 0 | 0.54 | 0 | 0 | 0 | 0 | 0.02 |
| 12 | Female | 519.225 | 616.08 | 612.26 | 23 | 23.22 | 23.365 | 26.12 | 12.635 | 15.71 | 24.335 | 18.06 | 21.395 |
| 13 | Female | 1193.635 | 1990.67 | 1639.495 | 0.56 | 1.79 | 2.4 | 1.07 | 2.53 | 4.16 | 7.985 | 3.82 | 3.93 |
| 14 | Female | 285.205 | 445.455 | 513.66 | 0.305 | 0.93 | 1.36 | 0.025 | 1.105 | 1.49 | 0.105 | 1.205 | 1.955 |
| 15 | Female | 2620.09 | 3778.31 | 3133.74 | 2.015 | 2.525 | 1.31 | 3.635 | 12.995 | 14.435 | 4.895 | 4.565 | 3.455 |
| 16 | Female | 1039.855 | 1922.345 | 1103.79 | 2.83 | 1.765 | 0.47 | 2.59 | 1.68 | 1.105 | 7.81 | 4.94 | 3.005 |
| 17 | Female | 2100.565 | 2842.775 | 3170.435 | 38.67 | 23.445 | 32.84 | 60.175 | 22.02 | 44.745 | 51.23 | 65.555 | 69.695 |
| 18 | Female | 1142.03 | 1741.705 | 768.335 | 4.04 | 5.885 | 6.08 | 12.53 | 2.4 | 2.22 | 6.27 | 8.685 | 5.025 |
| 19 | Female | 1254.575 | 1435.915 | 977.58 | 24.995 | 6.635 | 0.71 | 2.055 | 2.425 | 0 | 18.69 | 11.06 | 8.305 |
| 20 | Female | 1692.86 | 1692.86 | 1868.23 | 7.32 | 2.99 | 5.255 | 3.12 | 3.08 | 2.62 | 18.645 | 13.99 | 18.31 |
