# Supplementary material for: Immunotoxicity Assessment of Rice-Derived Recombinant Human Serum Albumin Using Human Peripheral Blood Mononuclear Cells
Source: PLoS One. 2014 Aug 6;9(8):e104426. doi: 10.1371/journal.pone.0104426 (PMC4123919; doi:10.1371/journal.pone.0104426)
Supplement: Table S4 — Individual result of IL-4 production. (DOCX) [file pone.0104426.s005.docx]

**Table S4.** Individual result of IL-4 production

| **Donor No.** | **Gender** | **PHA** | | | **PBS** | | | **pHSA** | | | **OsrHSA** | | |
| --- | --- | --- | --- | --- | --- | --- | --- | --- | --- | --- | --- | --- | --- |
|  |  | **24h** | **48h** | **72h** | **24h** | **48h** | **72h** | **24h** | **48h** | **72h** | **24h** | **48h** | **72h** |
| 1 | Male | 41.045 | 0.77 | 0 | 0.21 | 0 | 0 | 0 | 0 | 0 | 0.465 | 0 | 0 |
| 2 | Male | 4.76 | 1.465 | 0 | 0 | 0.375 | 0 | 0 | 0.465 | 0 | 0 | 0.405 | 0 |
| 3 | Male | 6.685 | 2.07 | 0 | 0 | 0 | 0 | 0 | 0 | 0 | 0 | 0 | 0 |
| 4 | Male | 6.42 | 0 | 0 | 0 | 0 | 0 | 0 | 0 | 0 | 0 | 0 | 0 |
| 5 | Male | 8.78 | 0 | 0 | 0 | 0 | 0 | 0 | 0 | 0 | 0 | 0 | 0 |
| 6 | Male | 8.595 | 0.45 | 0 | 0 | 0 | 0 | 0 | 0 | 0 | 0 | 0 | 0 |
| 7 | Male | 38.67 | 5.83 | 1.47 | 0 | 0 | 0 | 0 | 0 | 0.39 | 0 | 0 | 0.415 |
| 8 | Male | 16.885 | 2.285 | 0 | 0 | 0 | 0 | 0 | 0 | 0 | 0 | 0 | 0 |
| 9 | Male | 3.72 | 0.045 | 0 | 0 | 0 | 0 | 0 | 0 | 0 | 0 | 0 | 0 |
| 10 | Male | 2.3 | 0 | 0 | 0 | 0 | 0 | 0 | 0 | 0 | 0 | 0 | 0 |
| 11 | Female | 16.58 | 0 | 0 | 0 | 0.28 | 0 | 0 | 0 | 0 | 0 | 0 | 0 |
| 12 | Female | 8.4 | 0.985 | 0.565 | 0.465 | 0 | 0.55 | 0.535 | 0.245 | 0.535 | 0.455 | 0.19 | 0.32 |
| 13 | Female | 28.57 | 2 | 0.165 | 0 | 0 | 0 | 0 | 0 | 0 | 0 | 0 | 0 |
| 14 | Female | 13.86 | 10.13 | 1.33 | 0 | 0 | 0 | 0 | 0 | 0 | 0 | 0 | 0 |
| 15 | Female | 28.74 | 1.675 | 0 | 0.02 | 0 | 0 | 0 | 0 | 0 | 0.02 | 0.055 | 0 |
| 16 | Female | 66.57 | 13.125 | 0 | 1.58 | 0 | 0 | 1.69 | 0.175 | 0 | 1.22 | 0.2 | 0 |
| 17 | Female | 33.89 | 2.24 | 0.11 | 0 | 0 | 0 | 0 | 0 | 0 | 0 | 0 | 0 |
| 18 | Female | 24.355 | 1.87 | 0 | 0 | 0.055 | 0 | 0 | 0 | 0 | 0 | 0 | 0 |
| 19 | Female | 6.035 | 0.415 | 0 | 0 | 0 | 0 | 0 | 0 | 0 | 0 | 0 | 0 |
| 20 | Female | 10.46 | 1.15 | 0 | 0 | 0 | 0 | 0 | 0 | 0 | 0 | 0 | 0 |
